# Applying a cognitive behavioural approach in COPD: views of respiratory professionals

**DOI:** 10.1183/23120541.00474-2025

**Published:** 2025-12-22

**Authors:** Sian Newton, Ratna Sohanpal, Anna Moore, Clarisse Dibao-Dina, Hilary Pinnock, Liz Steed, Vari Wileman, Stephanie J.C. Taylor, Moira J. Kelly

**Affiliations:** 1 Wolfson Institute of Population Health, Queen Mary University of London, London, UK; 2 Barts Health NHS Trust, London, UK; 3 Faculté de médecine de Tours, Université de Tours, Tours, France; 4 Allergy and Respiratory Research Group, University of Edinburgh, Edinburgh, UK; 5 Health Psychology, Institute of Psychiatry, Psychology and Neuroscience, King's College London, London, UK

## Abstract

**Rationale:**

COPD is a long-term condition with comorbidities such as anxiety and depression that are associated with poorer health outcomes. The TANDEM (Tailored intervention for Anxiety and Depression Management in COPD) trial investigated whether a psychological intervention, delivered by respiratory healthcare professionals (“facilitators”) using a tailored cognitive behavioural approach, would reduce anxiety and/or depression and improve pulmonary rehabilitation attendance in patients with advanced COPD. The intervention did not reduce anxiety or depression or improve pulmonary rehabilitation attendance. As part of the trial process evaluation, we explored facilitators’ communication with patients, professional role and identity, and perspectives on implementation.

**Methods:**

Qualitative interviews with 14 facilitators (nurses, physiotherapists and occupational therapists) who were trained to deliver the cognitive behavioural approach sessions.

**Results:**

Facilitators recognised the need for psychological care and were positive about the training, practice and clinical supervision. The intervention was viewed as helpful for some, but not all, patients. Comorbidities and social and personal challenges affected patient engagement in identifying and addressing COPD-related anxiety and depression. There was scepticism about incorporating a cognitive behavioural approach intervention such as TANDEM into routine healthcare due to resource constraints.

**Conclusions:**

Respiratory healthcare professionals valued being trained and supported to deliver a tailored intervention using a cognitive behavioural approach to patients with COPD. The complexity of comorbidity and patients’ social context presented barriers to engagement. The intervention was viewed as unlikely to be delivered as part of routine care. Greater focus on patient engagement in the context of multimorbidity and psychosocial complexity is recommended.

## Shareable abstract

**Respiratory professionals valued being trained to deliver a cognitive behavioural approach for anxiety and depression in COPD. Comorbidities and psychosocial challenges affected patient engagement in addressing COPD-related anxiety and depression.** https://bit.ly/45e5PBL

## Introduction

COPD is a complex chronic disease associated with comorbidities that are under-recognised and undertreated [1]. Anxiety and depression are common [2, 3] (with mean prevalence of 36% and 40%, respectively [4]), negatively affect COPD management [5], and are often combined with difficult personal and social circumstances [6, 7]. Pulmonary rehabilitation is effective in improving physical health outcomes, including symptoms of anxiety and depression, for people living with COPD, but attendance and completion rates are suboptimal [8]. Anxiety and depression may be barriers to pulmonary rehabilitation attendance [3]. Psychological interventions, such as cognitive behavioural therapy, can potentially reduce anxiety and depression in people with COPD, indicating benefits of a more holistic care approach for patient self-management and well-being; however, further research is needed [3, 9–12].

As part of the process evaluation of the Tailored Intervention for Anxiety and Depression Management in COPD (TANDEM) randomised controlled trial, we investigated the experiences and perspectives of respiratory healthcare professionals on being trained in, and delivering, a tailored intervention using a cognitive behavioural approach to people living with COPD and anxiety and/or depression.

### The TANDEM trial

The TANDEM trial investigated the effectiveness of a cognitive behavioural approach intervention for mild–moderate anxiety and/or depression in people living with moderate–very severe COPD [13]. Participants were eligible to attend an assessment at their local pulmonary rehabilitation service at the time of randomisation. The TANDEM intervention was designed to take place before pulmonary rehabilitation attendance, between referral for pulmonary rehabilitation assessment and the start of a course [13, 14]. Patient participant characteristics can be seen in Taylor *et al.* [14]. Respiratory healthcare professionals (“facilitators”) were recruited and trained to deliver the intervention to patients over six to eight sessions at home or at a local clinic setting, with the support of a manual and supervision, and tailored to the patient's level of anxiety and/or depression. The minimal dose of intervention was the first two sessions. The intervention aimed to reduce anxiety and/or depression by changing patients’ cognitions and behaviours, based on a programme theory [15]. The cognitive behavioural approach applied in TANDEM was developed as a low-intensity form of cognitive behavioural therapy, built on the premise that how an individual thinks about their COPD influences how they behave and feel. Central to the cognitive behavioural approach is a “formulation”, which identifies and explains the interconnection between thoughts, feelings, physical sensations, and behaviour, and how unhelpful thoughts and behaviours may trap people in a negative cycle, affecting how they experience and manage their condition. The cognitive behavioural approach used in TANDEM aimed primarily to address negative automatic thoughts but not engrained thinking patterns. It drew on a defined range of techniques that could be taught to respiratory healthcare professionals without formal psychological qualifications. Our decision to use this approach in TANDEM was informed by the aim of enhancing routine clinical care through a psychologically informed intervention that could be integrated into the usual clinical roles of respiratory healthcare professionals. The intervention did not reduce levels of anxiety and depression in those who received it compared with controls [14].

Facilitators were recruited through social media and professional networks. They underwent a formal interview with members of the research team before undertaking a 3-day training programme, followed by a competency assessment through video-based role play with a simulated patient actor. Facilitators were funded separately for the training and intervention, apart from their main clinical roles. They received fortnightly clinical supervision (by telephone) from qualified cognitive behavioural therapists. The structure and content of the sessions can be seen in table 1.

**TABLE 1:** TANDEM session content

	Topics covered	Content
**Session 1**	Introduction, setting expectations	
	Topic 1: What is COPD?	Eliciting the patient's understanding of COPD
	Topic 2: Taking control of COPD	Identifying and working with illness and treatment beliefs and acceptance
	Topic 3: The patient experience of breathlessness	Teaching basic breathing control
**Session 2**	Topic 4: Introducing mood and COPD	Conducting a formulation and presentation of a cognitive behavioural approach
**Sessions 3–7**	Topic 5: Managing anxiety and COPD	Up to four sessions to conduct cognitive behavioural work on anxiety and/or depression dependent on individual need
	Topic 6: Managing depression and COPD	
	Topic 7: Applying the cognitive behavioural approach to other problems (optional)	
	Topic 8: Living with COPD day to day	Self-management approaches to COPD
		Learning to problem-solve and set goals
**Final session**	Topic 9: Preparing for pulmonary rehabilitation	Expectations of pulmonary rehabilitation, addressing worries and concerns

Ethical considerations regarding patient care and safety were carefully considered. Inclusion and exclusion criteria for patient participants are reported in the main trial papers [13, 14]. Patients with severe anxiety or depression as assessed by the Hospital Anxiety and Depression Scale (with scores ≥8 to ≤15 for one or both conditions) [16] were not eligible for the study. If facilitators felt that the patient would benefit from further psychological care after the intervention, they wrote a discharge letter to the participant's general practitioner. Safeguarding protocols (*e.g.*, actions if a patient mentioned suicidal thoughts and processes for continuing or stopping intervention delivery) received ethics approval and were included in the facilitators’ manual.

The TANDEM process evaluation aimed to describe and understand the processes by which the trial was conducted and to consider their effect on trial outcomes [17]. Qualitative evaluation of facilitator perspectives aimed to explore experiences and perspectives regarding the intervention. The study research objectives are shown in table 2.

**TABLE 2:** Study objectives

To explore the experiences and perspectives of TANDEM facilitators regarding the intervention and post-trial implementation.
To assess the acceptability of the intervention to facilitators delivering the intervention regarding: Patient-facing cognitive behavioural approach sessions Facilitator training Management of workload Supervision
To explore facilitators’ views about the potential impact of the TANDEM intervention on health and quality of care.
To identify the barriers and facilitators to implementation.
To identify and explore any unexpected consequences.

## Methods

The study working group included a core team (S.N., M.J.K., A.M. and R.S.) who met to design, discuss, and review methods, materials and data analysis, with multidisciplinary members of the process evaluation team (L.S., H.P., S.J.C.T., V.W. and C.D.-D.) advising and assisting with design and interpretation of the findings. Our research approach was reflexive and iterative, applying a generic qualitative design [18, 19]. The study topic guide (outlined in table 3) included questions related to the study aim and objectives. The study was approved by the London-Queen Square Research Ethics Committee, reference 17/LO/0095.

**TABLE 3:** Interview topic guide

**Participation in TANDEM** Motivation to participate in TANDEM
**Cognitive behavioural approach sessions with patients** Experiences of conducting cognitive behavioural approach sessions What went well Challenges Patient engagement Patient experience of the cognitive behavioural approach sessions Homework Logistics
**Training sessions** Training content Organisation of training Assessment Preparedness to deliver the intervention
**Supervision** Experience of supervision sessions Aspects that were helpful Aspects that were less helpful Logistics
**Professional identity** Feedback on participation in TANDEM Effect on role as respiratory healthcare professional Feasibility of using a cognitive behavioural approach with patients with COPD Confidence in using a cognitive behavioural approach with patients with COPD
**Post-trial implementation** Value of TANDEM as part of COPD care provision Feasibility of TANDEM as part of usual care Challenges to implementation

### Data collection

Qualitative interviews were conducted by telephone after the intervention phase of TANDEM, between June 2019 and April 2020, by S.N. and A.M. A total of 31 trained facilitators were deemed competent to deliver the intervention and approved to proceed with the trial. Details of how competence was assessed are reported in Wileman *et al.* [20]. For practical reasons unrelated to the trial, three facilitators did not deliver any sessions, leaving 28 facilitators to implement the intervention and thus eligible for interview. The 28 facilitators who delivered the intervention (seven nurses, 14 physiotherapists, six occupational therapists and one physiologist) were degree educated and employed within the UK National Health Service at a senior level: senior/specialist band (n=6), advanced management band 7 (n=17) and senior management/clinical lead band 8 (n=5). None of the facilitators had received previous formal training in cognitive behavioural therapy, but all had expressed interest in taking a holistic approach and learning more cognitive and behavioural approaches. Written consent was given before the qualitative interview. Interviewees and interviewers were not aware of preliminary or final results.

Sampling was purposive [21], with sampling criteria being the number of patients seen and professional group. A total of 14 facilitators were interviewed: five were nurses, five physiotherapists and four were occupational therapists. Nine interviewees had delivered the intervention to five or more patients, and five interviewees delivered the intervention to four or less patients. Interviews were audio-recorded, transcribed verbatim and all identifiers were removed before being stored securely for analysis.

### Data analysis

Our approach to analysis was informed by qualitative research principles, in particular analytic induction (core themes are identified through data analysis) and constant comparison (core themes are checked across the data to ensure validity and reliability) [22]. Transcripts were discussed throughout data collection so that the topic guide could be adapted to include unanticipated issues. A coding framework (supplementary table S1) was developed by the core team who each read two transcripts to identify coding categories, which were then discussed and agreed. NVivo (version 12) was used to organise data and assist analysis. Consistency in the application of the coding framework was reviewed by the core team.

We aimed both to describe and to interpret the data through a process of abstraction and recontextualisation [23]. Data were abstracted during coding of the whole dataset to reduce bias and avoid pre-empting themes. After data coding using NVivo, the coding categories were summarised and discussed. This was followed by recontextualisation by going through the process of interpreting the data, leading to the identification of three key themes. Refinement of themes was undertaken in a workshop with the wider working group. We then referred back to our research aim and reviewed the findings in relation to the literature.

## Results

### Engaging patients in a psychological approach

TANDEM was viewed by facilitators as a whole-person approach, incorporating body and mind, allowing them to build a cognitive behavioural understanding of the patient's day-to-day challenges. It enabled both parties to understand the thoughts that might be influencing behaviours and experiences of COPD symptoms, and how these linked to mood (table 4). Many patients reported that spending time talking about experiences of COPD and exploring feelings was a new and valued experience. However, early cognitive behavioural approach sessions were found to be demanding, as the shift to patients identifying and exploring relevant areas to work on was new to most facilitators and patients. Time was needed to build rapport and orientate to the cognitive behavioural approach.

**TABLE 4:** Data extracts on patient engagement

**Building rapport**	“On the initial session I probably spent more time getting to know the patients and finding out about their COPD… because I felt I needed to get to know the patient themselves on that first session and to develop that rapport with them so that then they would feel that they were listened to, and they could open up and talk to me about things” (physiotherapist 1).
	“In the hospital we're just literally focusing on their COPD. You've not got the time to be looking at this person as a whole and seeing how things link, or you don't ask them anything about… You don't ask them about stresses or anything like that” (occupational therapist 1).
	“His feedback afterwards was like he really valued TANDEM because he'd never had the opportunity to sit down and talk to a respiratory clinician one-to-one and ask questions, and to really understand his disease. …and because it's so patient-centred, if you're doing it right it's about asking them and them leading it” (occupational therapist 2).
**Talking about COPD symptoms**	“While all we'd really done was talk about it and allow him to open up about being scared about being breathless, by him talking about it kind of changed his whole perception of it and he realised that he could do more. Without even setting small goals he kind of just moved on in leaps and bounds and was getting on public transport on his own” (nurse 1).
**Multimorbidity**	“He did get breathless, but his anxiety wasn't due to his breathlessness. His anxiety was due to his pain. He's a man who's got chronic pain and he's had several operations on his back, on his fingers, on his arms to help with his pain. I did discuss that with my supervisor, and she said just target it at his pain, because that's obviously his trigger” (nurse 3).
**Complexity of patients’ lives**	“And I don't know whether it's just the patients that I've had, but one, her daughter's been in hospital. Another one, she's just lost her brother and now her niece has been diagnosed with cancer. Another one, she's got a poorly elderly aunt who she's had to move in with. Actually they've just got really busy lives, and we're already asking them to take at least an hour out each week to participate in the intervention sessions. And a lot of them I should think find that addition of them having probably half an hour's homework at least, it's a little bit too much” (physiotherapist 2).
	“They have got so much to say and so many issues. And it's quite hard to then say well, I'm here for your COPD. Because everything links, doesn't it? It's like…it's quite hard to just look at that” (occupational therapist 1).
**Timing of intervention**	“I'm not sure whether we're catching people maybe too late some of the time. I felt that a lot of the behaviours that were perhaps making them feel low in mood had been engrained for so many years that actually it was quite… I found it quite challenging in a short period of time to actually help them make change positively” (physiotherapist 1).
	“I think the younger patients were able to engage a bit more. I think there was more motivation for them. They had perhaps younger grandchildren or were still out and about in the community or were just on the tipping point of it becoming difficult for them” (physiotherapist 3).

Facilitators reported that patient experiences of COPD symptoms varied. Patients did not necessarily identify themselves as anxious or depressed, so the characteristics and relationships between anxiety, depression and breathlessness needed to be explored to break down and enhance their understanding of COPD, how it affected them and how symptoms could be better managed. By discussing activities and experiences, some patients realised that fear and anxiety could be associated with breathlessness, and could in turn be addressed.

Protected time was reported to be key to exploring patient concerns and supporting problem-solving. Patients valued the time committed by a respiratory healthcare professional to listen and help them understand their illness experience. Patients were reported to be more comfortable discussing psychological or social concerns at home than in a clinic setting, where the primary focus is more on the clinical management of COPD.

Most facilitators reported variability in terms of patient benefits. The intervention was seen to work well for some, but not all, patients for a variety of reasons, such as patients being content with their situation, experiencing psychological deterioration since recruitment, feeling lonely or finding it difficult to articulate emotions. The ways in which the complexity of patients’ lives emerged during the cognitive behavioural approach sessions were observed to influence their experiences of COPD and individual responses to it. Formulation techniques elicited psychological, physical and social issues that were interconnected, making it difficult at times to focus specifically on anxiety and depression related to COPD. Multiple issues regularly arose that were often not related to breathlessness or COPD, including comorbidities, severe mental health problems and the health of family members. TANDEM was structured around anxiety and depression related to COPD, so keeping sessions “on topic” was difficult at times. Some facilitators commented that patients required tailored support to address other problems before committing to interventions aimed at improving anxiety or depression related to COPD.

The many life challenges patients faced were thought to have affected their home practice of psychological techniques between sessions. Some facilitators commented that it was more difficult to motivate patients with severe COPD due to the difficulty of changing behaviour, mood and expectations that have become engrained over many years. Using a cognitive behavioural approach with younger patients and those diagnosed more recently was viewed as likely to be more successful. In contrast, some facilitators described older patients who were extremely receptive throughout the sessions.

### Professional role

A primary reason for facilitators to participate in TANDEM was to enhance skills and improve the physical and psychological health of their patients (table 5). COPD was perceived to entail a huge psychological component to the symptom burden, with anxiety and depression considered to be underdiagnosed and undertreated. TANDEM was seen as a practical approach to addressing these issues.

**TABLE 5:** Data extracts on professional role

**Training**	“It was kind of stressful during training to be videoed doing this thing for the first time with somebody who was potentially what a real patient would present like in terms of the level of anxiety and depression that they were demonstrating… it was great that they made it so difficult, because I have had participants who have been as challenging as that, and so it did help to feel that I was on the right track” (nurse 2).
**Enhanced skills**	“I think I've got a lot more tools in my toolkit for being much more of a holistic practitioner in terms of my knowledge around the psychosocial side. What I was doing was limited in terms that I could recognise yellow flags, psychosocial barriers to treatment being effective, but it's having the skills to try and help manage that. It was always a case of referring on in the past” (physiotherapist 5).
	“Initially I was finding it really hard to step away from giving solutions and giving physical responses to patients. Now I am just allowing them to open up a bit more and talk about how they're feeling and what their solutions might be. And I think it's massively changed my practice in that respect for the better I think” (nurse 1).
	“The cognitive, I was always a bit nervous about because I'm very much a nurse, a doer, and I worked for a helpline for four years and it took me a while to really get used to listening and pushing it back to someone else to make their decisions rather than take it from them and crack on and organise it for them” (nurse 5).
**Mental health challenges**	“I think I felt a bit out of my depth in terms of different health conditions. I've worked in different areas, so that's fine, if they had a bad back limiting them and things like that. But with the mental health side, because I've not actually treated patients with severe mental health problems, I found that quite challenging” (occupational therapist 1).
**Clinical supervision**	“And I think it's probably just what struck me is how complicated everyone's lives are. And I've just been really grateful for the supervision, to keep everything on track and to point out that actually, these patients’ lives are complex and you're not going to be able to sort everything out with them” (physiotherapist 2).

All professional groups interviewed reported developing new and transferable skills in psychosocial aspects of respiratory care and specific skills in working with anxiety and depression. The training was found to be rewarding and positively challenging (especially the video assessment), adding depth to communication skills, particularly listening and exploratory questioning. Learning through practice and feedback in a group with peers improved confidence in what was viewed as a highly skilled role. Some described developing “literacy” in mental health.

The TANDEM structure provided space to improve understanding of patient day-to-day COPD management. Facilitators described taking a step back and supporting patients to identify problems and explore ways of addressing them independently. Resisting the urge to offer clinician-led solutions was a significant behaviour change for many. Facilitators found themselves having difficult conversations, including navigating through complex medical histories or life events, to identify causes of anxiety and/or depression. Several facilitators questioned the suitability of some patients for the intervention when other conditions were potentially quite severe, particularly if they had other mental health conditions.

Clinical supervision was considered to have contributed to confidence and competence in using a cognitive behavioural approach. It was also viewed as necessary when taking on “someone's emotional burden” (nurse 5) and negotiating difficult conversations. It could be difficult to stay “on script” during sessions, and having a supervisor proved to be a valuable resource, providing guidance and clarity on what to prioritise and how to navigate complex discussions effectively.

### Does psychological care by respiratory healthcare professionals have a place in standard COPD care?

Facilitators believed in the value of TANDEM and stated that it should be available as part of respiratory care (table 6). They also commented that, with appropriate training and supervision, they were able to deliver a cognitive behavioural approach. However, they foresaw problems for implementation due to resource and cultural constraints, including pull-back to usual professional roles within their respective teams. Facilitators stated that protected time to see patients was important and it would not be possible to undertake a structured cognitive behavioural intervention as part of their usual clinical post. For TANDEM to be implemented in clinical care, wider strategic and cultural changes were considered necessary within the organisational context of COPD care. There was a discourse of “limited resources” across the interviews, with facilitators advocating for the inclusion of psychological interventions in COPD care, while simultaneously expressing scepticism about their viability. Convincing commissioners was viewed as a significant barrier to implementation.

**TABLE 6:** Data extracts on intervention implementation

“The opportunity to focus more on the aspects of treating patients that were always time pressured and knowing that actually taking the time to do those, what we consider softer sides of input actually made a difference to the outcomes. And the opportunity to try and gather evidence to prove it actually made a difference, and that it's worth investing in” (physiotherapist 5).
“I guess training and turnover of staff might be an issue, because I'm thinking of my own experience, I think if that was my only role I would miss my other role. So, it's been quite nice for me to have it just one day a week and then do the rest of my role the rest of the week” (physiotherapist 4).
“…it will depend on where… which teams are taking it on because I think if people are using it as an add on to what they have it means everything takes longer because they're doing more things, if that makes sense. If you've got somebody that this is their role then it's great, because they can just use this and really make a difference” (occupational therapist 4).
“It's a lovely thing that we're doing, but do I think in reality it will carry on? I'm not sure, because of funding if I'm honest. I think funding will be a big issue” (nurse 4).

## Discussion

Training and supervised practice in a structured cognitive behavioural approach for people living with COPD and anxiety and/or depression was valued by TANDEM facilitators. It was considered to enhance clinical practice and to benefit some patients. However, developing a collaborative relationship with patients was viewed as challenging, especially in the early cognitive behavioural approach sessions. Focusing on COPD content, such as breathlessness, presented difficulties, as facilitators found that session topics opened up many interconnected health and psychosocial issues.

Collaboration between patients and clinicians is highlighted as a principle of care for people living with COPD [12], with mutually respectful and regular relationships with respiratory healthcare professionals found to empower and facilitate patients to better manage their disease [24]. However, whereas self-management is endorsed by respiratory healthcare professionals in principle, the shift to sharing control is a difficult process in practice [25–27]. In turn, although patients with COPD value personalised, empathic care, they report difficulty in communicating holistic support needs to healthcare professionals [25]. Facilitators described gaining confidence to support patients in taking an active role, identifying goals meaningful to them and exploring ways to address these goals. Patient participants in TANDEM reported positive experiences of relationships with facilitators, valuing the opportunity to reflect on their COPD and make connections between physical and mental health [28]. At the same time, this process was often experienced as demanding for both parties. Overall rapport was viewed as good; however, the shift to a truly therapeutic alliance [29], with the patient being the main agent of change, was challenging.

Analysis of session audio-recordings in the TANDEM fidelity evaluation showed that facilitators delivered the intervention with acceptable therapeutic competency [20]. However, although agendas were set, the patient was only involved in agenda setting in 30% of cases examined. This may have been driven by TANDEM being manualised with a clear focus on mood in relation to COPD, but it suggests that a fuller understanding and agreement with patients about whether TANDEM meets their needs and priorities, at the time of the offer, is important. It is also possible that patient engagement with the cognitive behavioural approach would gradually become more effective, as facilitators reported gaining confidence with experience, corresponding with the fidelity analysis finding that therapeutic competence increased over time [20].

The complexity of the lives of patients with COPD was evident in the interviews, which is in line with earlier research [4, 6, 7, 30]. However, the extent to which patient engagement unfolds within the context of complexity, creating a potential barrier to the cognitive behavioural approach's process of change, was greater than expected. People living with chronic illness may present accounts of life where illness is present but not foregrounded [31]. Patients with COPD have been found to normalise disabilities, negating the perceived need for assistance with symptoms [32]. Interviews with TANDEM patient participants [28] showed that many had established coping strategies such as stoicism, having adjusted their sense of self over the years to fit limitations associated with COPD. COPD was present but not always the central concern at the time of the trial, impacting on development of a therapeutic alliance around addressing anxiety and depression associated with COPD symptoms. In the context of living with COPD, other significant comorbidity and psychosocial issues appeared to override COPD-related concerns. The mental health and social issues that arose were often long-standing and at times related to trauma, as found in previous research [4]. In this situation, more in-depth therapy, with COPD as an element but not the primary focus, is likely to be needed.

Facilitators reported that TANDEM did appear to benefit patients who linked their anxiety and/or depression to their COPD symptoms. However, not all patients identified themselves as depressed and/or anxious. In these situations, facilitators observed increased personal awareness of mood through the TANDEM sessions, with patients going on to develop cognitive and behavioural techniques to better manage their symptoms. Given that 41% of TANDEM patient participants had attended a pulmonary rehabilitation course in the past, it is possible that this may have been one factor influencing their baseline understanding of links between mood, behaviour, thoughts and symptoms.

The Hospital Anxiety and Depression Scale was used as a generic measure of anxiety and depression in the TANDEM trial. However, our qualitative study indicates that assessment tools specifically designed to identify COPD-related anxiety and depression may have been preferable [3, 5, 33]. Future use of such tools may better reach those who would most benefit from disease-specific interventions [34] such as TANDEM. The timing of cognitive behavioural interventions appears to be important, raising the question of how best to identify and deliver appropriate psychosocial interventions for patients at the right time for them. Intervening earlier in the disease trajectory to address anxiety and depression as they arise, before they become entrenched, may improve the outcomes of such approaches. Research is needed to both develop effective assessment measures and implement stepped-care strategies [35].

Respiratory care stakeholders support the need for psychological interventions such as TANDEM for COPD but raise concerns about the resources required [36]. Interviewees did not think it was feasible to undertake a psychological intervention such as TANDEM in their usual clinical posts due to resource and structural constraints, primarily funding and time. The discourse of limited resources may be a potential barrier to innovative psychological interventions.

Education, training and support for respiratory healthcare professionals to explore psychosocial needs, assist patients with COPD self-management and support empowerment is advocated [9, 37, 38]. The acceptability of the TANDEM model to respiratory healthcare professionals indicates that it can usefully inform future training and practice. All interviewees reported gaining transferable skills in patient-centred and psychological care. Clinical supervision is viewed as a necessary support for clinicians delivering psychological care [39] and was strongly endorsed by facilitators. There is a need to fine-tune skills in patient engagement to overcome barriers to involving the patient as the main agent of change, particularly in agenda setting, as noted elsewhere [37].

Our study aids understanding of why the TANDEM intervention did not reduce anxiety and depression in this population of people living with COPD, through an in-depth exploration of the views and experiences of facilitators. However, there were a number of limitations. Facilitators were a self-selected group of senior clinicians who were motivated to develop better awareness of mental health and provide holistic care. We do not know what proportion of respiratory healthcare professionals share these perspectives. While half of all facilitators were interviewed, those who were not interviewed may have different views of the intervention. Given the limited number of facilitators interviewed, we have not made comparisons between facilitator characteristics, such as clinical role.

Several implications have been identified. Psychosocial care that can be matched to patient need at the time of delivery is needed as part of a stepped-care approach. This requires improved screening and assessment tools to identify anxiety and depression related to breathlessness. The value of training for respiratory healthcare professionals in low-intensity psychological approaches should be further assessed, with an emphasis on patient engagement. Further consideration of how to integrate low-intensity psychological therapy into routine respiratory care is needed.

### Conclusion

Training and supervised practice in psychological care for people living with COPD using a cognitive behavioural approach was valued by respiratory healthcare professionals and considered to enhance clinical practice and benefit some patients. Additional embedding of patient agenda setting in facilitator training and support is indicated. The complexity of patients’ lives appeared to impact the likelihood of a cognitive behavioural intervention positively affecting clinical outcomes in anxiety and depression. Improved assessment of patient suitability for psychological interventions targeting anxiety and depression related to COPD is warranted.

## Supplementary material

**Please note:** supplementary material is not edited by the Editorial Office, and is uploaded as it has been supplied by the author. Supplementary material 00474-2025.SUPPLEMENT
