# Supplementary material for: Applying a cognitive behavioural approach in COPD: views of respiratory professionals
Source: ERJ Open Res. 2025 Dec 22;11(6):00474-2025. doi: 10.1183/23120541.00474-2025 (PMC12741843; doi:10.1183/23120541.00474-2025)
Supplement: Supplementary file 1 [file 00474-2025.SUPPLEMENT.pdf]

Table S1 – Data coding category framework

CODES	SUB CODES	DESCRIPTION	DATA EXAMPLE
**Communicating with patient participants**			
	Building rapport	Facilitator views on building rapport with participant	*On the initial session I probably spent more time getting to know the patients. And actually finding out about their COPD, rather than getting onto doing hot cross buns and spidergrams of breathlessness, which I often then tried to come to in session two really, because I felt I needed to get to know the patient themselves on that first session.*
	Carers	Views on carers being present, advantages/disadvantages	*There was one carer that sat in the session, which was good because then she was able to… This is the lady that really struggled to go out of the house. So she was very much trying to push her, but it was good for me to say its not going to be a quick fix.*
	Challenges	NEW CODE - similar to existing node: TANDEM Involvement\Involvement challenges	*The difficult thing was initially trying to explain to study participants what I would be doing and why I would be doing it when it was also something that I was getting to grips with*.
	Homework	Comments on the participant's homework	*So after the first session we always start with the home practice at the beginning of the next session. So how did you find it, did you find it useful, have you got any questions about it.*

			Can I look at what you've done? Can we talk it through?
	Participant engagement	How Facilitators found engaging with participants; difficult, easy, skills used, did some engage easier than others	*They can be a bit reserved at first, because they're not sure what it's about. And then usually by week two we've broken the ice a bit. And they're just interested.*
	Participant understanding of breathlessness	Expectations of the intervention/CBA. views on TANDEM	*And so I'm finding that a bit of a struggle, because I seem to always be bringing it back to his breathlessness because that's my role. And I'm trying to make it a bit more loose and just kind of bringing it back to the context of living with a long-term condition. Because it's not really his breathlessness, and he's even challenged it.*
	Silences	How facilitators found the use of silences in TANDEM/in general	*I did feel like I was doing a lot of talking and looking back at…I probably should've left…it doesn't matter if there was a silence or if the person needed to think a bit more.*
	Value of space to talk	Patient participants comments on value of the space to talk about their condition, breathlessness or in general	*I think they like the fact that someone's there supporting them. They felt very much like they're being listened to.*
**TANDEM content delivery**			
	Implementation fidelity	NEW CODE - Facilitators comments on how well they kept to the order of delivery, if they changed anything.	*But just sometimes things would come up and I would just…because of my own clinical reasoning I think well, actually, he needs a bath board. It wouldn't be right for me to start doing a*

			hot cross bun on him or breathlessness management.
	Value to patients of the intervention	NEW CODE – perceptions of value to patients of TANDEM	*So thinking along…identifying those patients who maybe only need six treatment sessions, and then discharging them, whereas previously I probably would have held onto them for a bit longer because of the chronic nature of their disease.*
**Mental health**		NEW CODE - learning how to deal with patients with mental health difficulties, dealing with mental health, lack of these skills prior to TANDEM or in HCW in general.	*I've worked in different areas, so that's fine, if they had a bad back limiting them and things like that. But with the mental health side, because I've not actually treated patients with severe mental health problems. I found that quite challenging.*
**Participant lives**			
	Day-to-day life	Day to day life for participants with a LTC e.g. challenges, hopes, feelings.	*I've been in primary care a long time, even with limited time to engage with patients. So, I had an inkling of how chaotic people's lives are.*
	Living with a long-term condition	Similar to node: Participant's lives\Day to day life	*And clearly people with a chronic condition have very complex…well, we all have very complex lives.*
	Complex lives	How complex these participant's lives can be e.g. with multimorbidities, other family members' health, finances etc.	*She'd got all sorts of social problems. So really her focus for being in the project was not particularly to do with breathlessness and COPD, but that she's got COPD. I think she just wanted somebody to talk to about all these problems.*
	Illness trajectory	Whether illness trajectory stage affects how	*I think the more severe the COPD was,*

	stage	a participant engages with CBA or other aspects of TANDEM.	*it was harder to get them motivated and get them out of the… Because I think we've talk about vicious cycles a lot, haven't we? I think that they'd been going along so many years and the same pattern and the same thoughts, it was hard to then change their approach to the condition and I think they were quite deconditioned by that point.*
	Multimorbidities	Comments on patients/participants with multimorbidities; low mood/anxiety, how TANDEM may or may not help, difficulties with engagement/adherence.	*There's a massive cohort of patients, and you think about the demographic group of patients with COPD, and they have lots of other life stresses that are happening. Usually they've got multiple comorbidities. And they might be carers, generally there's financial issues, carer issues, social issues. And I was just saying that because they have all these other barriers to self-management in the broader…not just exercise, but the broader way of conceptualising self-management, that those barriers need to be addressed.*
	Self-management	How participants manage their condition(s); behaviours, exercise.	*Suddenly the penny dropped and she actually started doing a bit more and starting setting herself little challenges, going out walking more and started sleeping in her bed again, instead of on the settee.*
	Talking about breathlessness	Facilitators comments on difficulties conversing due to breathlessness, participants' views on their breathlessness.	*And so I'm finding that a bit of a struggle, because I seem to always be bringing it back to his breathlessness*

			because that's my role. And I'm trying to make it a bit more loose and just kind of bringing it back to the context of living with a long-term condition. Because it's not really his breathlessness, and he's even challenged it.
**Professional practice**			
	Change in practice role	For example, being a physio, nurse... problem-solving approach. Any changes seen in their current role since being in TANDEM. Similar to another existing node: Professional practice\Impact on professional ID	*I think I've got a lot more tools in my toolkit for being much more of a holistic practitioner in terms of my knowledge around the psychosocial side of what I was doing was limited in terms that I could recognise yellow flags, psychosocial barriers to treatment being effective.*
	Continuing development	Regarding the continuation of their professional development	*I've done a little bit of motivational interviewing just on previous courses. But it was nice to actually spend the time to actually look at that in my detail and have a little bit more of an understanding about how to manage individual patients on a one-to-one basis, and actually make it personalised to them.*
	Impact on professional identity	Similar to existing node: Professional practice\Change in practice role	*As a physiotherapist I'm often facilitating people to be, I'm often looking at practical solutions. So for me to actually take a step back and be quiet, a try and facilitate the person to look at how they could reach those*

			solutions, that was quite different.
	Professional identity	Information about their current role	*It's given me confidence and it's given me confidence to articulate myself in terms of my professional identity and what I can offer. Because often people don't understand the role of respiratory occupational therapists and what we can add.*
	Professional practice challenges	Challenges in their current role/services. Includes practicalities	*You don’t get many occupational therapists working in a respiratory team so most of the time it’s just one, I was the only one in that team and I’m the only one in the team I’m in now. So… and I think it just depends on where you work and what other people feel an occupational therapist can do because sometimes if your managers don’t understand what we can do, put in to discharge planning or equipment or transfers, they don’t always see the opportunity of where we can work and make a difference.*
	Using TANDEM skills in professional practice	Using skills learnt in TANDEM outside of TANDEM capacity	*The questioning, the socractic questioning, and trying to drill it down why, why, why. Why do you say that? Why do you feel that? Because that's maybe something, well, it wasn't something that I did before…to the extent that I'm doing now. I did do it, but not to the extent I'm doing it, if that makes sense. And maybe I feel more confident having had the training and*

			delivering this particular study that that's what's needed to actually ascertain what the real issue is.
**Pulmonary rehabilitation**		NEW CODE - anything regarding PR	*So previously in my role we would identify patients using a GAD-7 or a PHQ-9, and if they had higher scores we would not enrol them pulmonary rehab, we'd refer them to the GP for input. So the opportunity to do things exactly in TANDEM together, supported and through PR but with that psychological support was a really interesting concept to look at.*
**TANDEM implementation**			
	Acceptability to other professionals	NEW CODE - similar to node TANDEM implementation\Non-TANDEM colleagues views	*In terms of the other OTs that I meet in [names forum], they were really interested. Really, really interested, and wanted to learn more about it. But I think the difficulty is lots of other respiratory colleagues, they're bogged down with their own work.*
	Community services	Anything regarding existing community services	*I can't see it being universally accepted in primary care, simply because, I mean I've worked in primary care for a long time and I've never seen it quite so demoralising.*
	Facilitator role title	What should the role be called?	*I don't think it should be a distinct role. I think it should become a part of the toolkit of the relevant professionals.*
	IAPT	Anything regarding IAPT services	*The IAPT services, they run in a different format which don't necessarily*

			always suit our patients.
	Implementation challenges	Any potential challenges to implementing TANDEM in usual clinical care	*And I think the biggest challenge really in terms of disseminating it is to persuade commissioners that the benefits are long term enough or cost effective enough to justify the fact that it's quite an intensive approach.*
	Measures	NEW CODE - facilitators views on the measures used within the trial e.g. GAD	*I was using GAD and PHQ before, but more sporadically. Whereas I just do it more routinely now.*
	Non-TANDEM colleagues' views	NEW CODE – similar to node TANDEM implementation\Acceptability to other practitioners	*And so there may need to be some cultural shift in thoughts about the holistic nature to embrace this side of things in a little bit more detail.*
	Organisational context or culture	How TANDEM may or may not fit in with specific organisation cultures. People's beliefs regarding CBA, or the way in which an organisation runs.	*I think there's quite a lot of scope for us to be using these techniques perhaps with a little bit of supervision or discussion with a psychologist. So exactly what that looks like I'm not sure. But yes, as I said, I think the main issue would be all the team having that training, having that time to embed it in practice and to use it and to share learning.*
	Telephone support sessions	NEW CODE - anything covering telephone support for participants	*I think often they're quite quick calls because often they're really positive and everything has gone well. And we try and tease out what challenges they've had.*
**TANDEM involvement**			
	Fears	For example, seeing first patient, delivering	*I think I felt a bit out of my depth in*

		CBA, feelings about dealing with participants with depression and anxiety	*terms of different health conditions. I've worked in different areas so that's fine. But with the mental health side, because I've not actually treated patients with severe mental health problems I found that quite challenging.*
	Hear about TANDEM	How the Facilitator heard about TANDEM	*When I was working with the respiratory team in [names place] one of my nurse colleagues met [name] at some event and she passed the information on to me.*
	Involvement challenges	Challenges in TANDEM such as being flexible around job, printing out documents, communicating the purpose of tandem, keeping to breathlessness	*It was just the practical things that at times were a little bit, you had to think ahead at all times, so that if I knew I had to print something else I could send it to myself at work to print, because then I'd have my three days at work then I'd do TANDEM on the fourth day and if I didn't have time in my normal workload it meant I was then without the handouts or what I needed for the tandem session*
	Motivation	Facilitator's motivation to take part in TANDEM	*We're a community respiratory service and we also run the rehab sessions from our team, our physios in the team run them. So I know that a lot of patients don't or can't complete rehab for lots of different reasons, but some for anxiety reasons. So I was interested to learn more about how I could help patients complete the programme.*

	Using TANDEM skills in TANDEM	NEW CODE - using specific TANDEM skills for/during TANDEM involvement	*I found that very hard in the beginning to take off my physio hat and come in with practical and physical solutions to problems.*
**TANDEM supervision**			
	Practicalities	For example, arranging phone calls with supervisor	*I think that it's useful to have that flexibility to contact… I mean there's obviously the usual times you would normally contact after sessions two and four, but to have the option to be contacting between specific cases and things like that has been useful.*
	Value	Value of the supervision	*It's a really good opportunity for me to just find out what else I could do differently and especially when I get a bit stuck and think what can I do about this. This is a bit challenging, how can I do this?*
**TANDEM training**			
	Feedback	For example, being videoed	*I think it'll be interesting to see how the different people had tackled different scenarios. Because it's not until you're in it… It's a bit like learning to drive, isn't it? It's not until you're actually in it that you actually realise what you didn't know, or what kind of issues sometimes came up, or common threads of challenges that sometimes came up.*
	Learning	Learning about anxiety and depression, how to talk to people about mental health, safety	*I think from a learning perspective I was aware already about anxiety and*

		and suicide	*depression, because that's something we're very aware of in pulmonary rehab. That's something we measure a lot. And also about learning. And I've done a little bit of motivational interviewing just on previous courses. But it was nice to actually spend the time to actually look at that in my detail and have a little bit more of an understanding about how to manage individual patients on a one-to-one basis, and actually make it personalised to them.*
	Preparedness	Feeling prepared to deliver intervention confidently	*Really helpful, really good. Very well developed, very thorough. The practice, the role-play, the videos, taping sessions was really good. And I think it really did help me develop confidence in it. I mean it was still really scary.*
	Quality/value	How Facilitators value the training; group experiences, handbook. Their views on the quality of the training.	*So the training was very good as well because they made us work through things on a personal level as well. And so then you could identify with how patients might actually feel. How you break problems down and how you look at things in a different way. And chatting to the others in the group, I know we all found that quite interesting.*
	Reflection	Reflections on the training	*And that I think was quite and continues to be quite a challenge. So the training very much obviously was focused on the breathlessness, that's*

			what we're there to help with. And then there was the session about applying it to other conditions.
	Resources	Manual, supporting paperwork	*I think the only thing to add, and now I've done it for a few months, is really talking me through the paperwork and what to fill in at what point.*
	Structure	Structure of the training	*I felt that the training was delivered in quite a methodical logical way. I think overall I felt that the training could've been a little bit longer.*

Explanatory note This table includes the coding categories initially identified by the research team in two interview transcripts. Further coding categories were identified during analysis and are added as 'NEW CODE'. The coding framework was applied using NVivo version 12. The term 'node' used in NVivo is called 'code' here.
